# Hepatitis C virus prevention and care for drug injectors: the French approach

**DOI:** 10.1186/s41124-018-0033-8

**Published:** 2018-06-05

**Authors:** Jean-Michel Delile, Victor de Ledinghen, Marie Jauffret-Roustide, Perrine Roux, Brigitte Reiller, Juliette Foucher, Daniel Dhumeaux

**Affiliations:** 1 Comité d’étude et d’information sur la drogue et les addictions (CEID), 20, place Pey-Berland, 33000 Bordeaux, France; 2 Hôpital du Haut-Lévêque, Pessac, France; 3 Cermes 3 (Inserm U988/CNRS UMR 8211/EHESS/Paris Descartes University) and French National Public Health Agency, Paris, France; 4 Inserm UMR1252/IRD/SESSTIM/Aix-Marseille University/ORS PACA, Marseille, France; 5 Université de Paris-Est, Créteil, France

**Keywords:** Drug users, Injection, Guidelines, HCV, PWID, Harm reduction, Treatment costs, Strategy, France

## Abstract

After France removed hepatitis C treatment reimbursement restrictions on 25 May 2016, an expert report presented recommendations, which focused on vulnerable groups including people who inject drugs (PWID). This commentary presents the key points of the chapter with a particular focus on policy.

Thanks to the official lifting of restrictions based on disease stage and to the excellent efficacy and tolerance of the new DAA (Direct-Acting Antivirals) among PWID, the main issue is to improve the HCV care cascade. In France, many HCV-infected PWID, especially active/current PWID, remain undiagnosed and unlinked to care. Our challenge is to improve HCV screening by point of care testing (POCT), outreach methods with mobile teams, rapid tests, FibroScan, etc. and to provide PWID with appropriate services in all the settings they attend, such as drug treatment or harm reduction services, social services, prisons, etc. Another important issue is the prevention of reinfection through comprehensive and long-term follow-up.

The report recommends a new national policy: testing and treating PWID as a priority, since this is the best way to eliminate HCV infection. It requires a global strategy consisting of combined and long-term interventions: prevention, outreach, screening, DAA, drug treatment programs including opiate substitution treatment (OST) and various harm reduction programs, including needle exchange programs (NEP). Ideally, these services should be delivered in the same place with an integrated approach. This should lead to meeting the national objective set by the government of eliminating hepatitis C by 2025.

## Hepatitis C virus prevention and care for drug injectors: the French approach

The hepatitis C virus (HCV) epidemic is a major public health concern [1–3]. In France, the number of HCV-infected people was estimated at 193,000 in 2011 [4]. Since then, more than 40,000 patients have benefited from the new treatments, the direct-acting antivirals (DAA), including 15,000 in 2016 and probably 20,000 in 2017. The number of patients still needing treatment is estimated at almost 115,000, of whom about 75,000 have not yet been tested [5]. PWID are the main focus of attention particularly in developed countries, where the prevalence of infection (antibody anti-HCV positive) is estimated to be 53% in Western Europe [6]. It is even around 65% in France so the problem is particularly acute [7]. PWID pay a heavy price for this infection in terms of mortality [8, 9] and they are the primary source of HCV transmission in France [4, 10].

## Background

For a long time, HCV treatments were not recommended for active PWID in France as in many other developed countries or they were deferred and challenged [11] for more or less explicit and acknowledged reasons. These included lack of concern and involvement of PWID, professionals’ concerns about the frequent psychiatric comorbidities, a particular intolerance to interferon as well as poor compliance with treatment [12] which is potentially worsened by social instability (absence of residence, isolation, etc.), and worries about reinfection [13]. However, very often treatment is deferred only because PWID feel stigmatized, as is also the case with HIV infection [14].

The introduction of the DAA, which are simple to take, effective and well-tolerated, has made it realistic to plan not only to cure most patients but also to control the HCV epidemic by reducing the risk of transmission [15, 16].

Given the limited impact on HCV transmission of current harm reduction interventions among PWID, it seems that access to DAA will prove efficient in decreasing HCV-related morbidity and mortality among PWID, as with HIV [17–19]. In combination with high coverage needle and syringe programs (HCNSP) and OST, treating infected people with DAA is the best way to prevent the spread of HCV and “to achieve substantial reductions (> 50 %) in HCV chronic prevalence over 10 years.” [15, 20, 21]. It would be feasible to envisage not only the elimination of HCV infection but also its potential eradication, an ail consistent with the concept of treatment as prevention (TasP) [22–25].

Indeed, modeling studies [26–29] have shown that HCV prevalence can be significantly reduced by treating PWID infected with the virus, whereas the impact of harm reduction interventions (OST or needle exchange programs, NEP) remains limited [30]. The TasP approach is also cost-effective [16]. The maximum effect is obtained by associating DAA and harm reduction interventions such as OST and NEP [21, 31].

With regard to DAA availability, Cousien et al. used a dynamic individual-based model including the PWID social network to simulate the impact of improved testing, linkage to care, adherence to treatment and modified treatment recommendations on the transmission and the morbidity of HCV infection in PWID in France. With the current incidence and cascade of care, HCV prevalence decreased from 42.8 to 24.9% (95% confidence interval: 24.8–24.9) after 10 years when treatment was initiated at fibrosis stage ≥F2. Changing treatment initiation criteria to treat from F0 was the only intervention leading to a substantial additional decrease in HCV prevalence, which fell to 11.6% (95% CI: 11.6–11.7) at 10 years. Combining this change with improved testing, linkage to care and adherence to treatment decreased HCV prevalence to 7.0% (95% CI: 7.0–7.1) at 10 years and avoided 15% (95% CI: 14–17) and 29% (95% CI: 28–30) of cirrhosis complications over 10 and 40 years, respectively [32, 33].

In this context, and following the first report on viral hepatitis [34] which concluded the third national action plan to fight HCV and HBV, the French Ministry of Health decided in May 2016 to grant universal access to DAA, which were until then reserved for patients presenting a high degree of fibrosis. The ministry commissioned a second report with updated recommendations for effectively reaching all affected people, especially the vulnerable groups like PWID [35]. PWID are an important population to target to improve HCV prevention using the TasP approach. To this end, the ANRS and the CNS were mandated to make recommendations to ensure that the most vulnerable groups and the furthest removed from healthcare services received the most adequate HCV therapeutic management. In 2014 and 2016, almost 200 clinicians, researchers, patients and non-profit organizations were mobilized to produce the recommendations drafted in these two reports.

Our contribution to the report was to coordinate the chapter for proposing recommendations on PWID and TasP.

### Organization of care and follow-up of HCV-positive drug users

Our group was composed of clinicians (hepatologists, addiction specialists, general practitioners, psychologists and social workers), as well as researchers (epidemiologists and social scientists), community representatives and drug users from self-support groups.^1^ To achieve our goal, we needed to identify the foreseeable difficulties and organize access to DAA for PWID in the most efficient manner. Interventions should be well adapted and all-encompassing to successfully engage and maintain PWID in treatment and care, as most people have psychiatric comorbidities and social vulnerabilities which make access to care and follow-up particularly challenging [13, 36]. Positive results often depend on personalized assistance [12]. To overcome these limitations, therapeutic progress should be accompanied by validated harm reduction interventions [21] that provide a real continuum of care. This was the basis for the working group’s recommendations, which are organized in four main topics: prevention, testing, treatment and an integrated approach.

### Drug use and healthcare provision: the French context

As defined by OEDT/EMCDDA (Observatoire Européen des Drogues et des Toxicomanies/European Monitoring Center for Drugs and Drug Addiction), problematic drug users are those who use opiates, cocaine, crack, amphetamines, and people on OST (opioid substitution treatment), as well as those who injected their drugs in the previous month. This is one of the five EMCDDA key indicators required in the European Union countries. France is in the higher half of the European Union countries for problematic drug use (Janssen & Bastianic, 2013) with an estimated 275,000 to 360,000 users (including 105,000 injectors) and a prevalence estimated at 7‰ and 9‰ (15 to 64 years old) [37]. Addiction care is mainly given in 430 specialized centers (CSAPA, Centre de soins, d’accompagnement et de prévention en addictologie) countrywide. These outpatient care services run by multidisciplinary teams treated around 280,000 people in 2014, including 80,000 for drug-related problems other than cannabis such as heroin and opiate abuse (Palle, 2016).

### Prevention

Interventions must address the risk of contamination by emphasizing prevention and harm reduction. In 2014 the number of PWID in France was estimated at 105000 with at least one injection during the previous year, including 85,000 active injectors, i.e. at least one injection during the previous month, including 35,770 who were monitored by a CSAPA in 2014. Of these, 27,674 were active injectors (Janssen, 2016). Some of the injectors who do not go to the CSAPAs go to harm reduction units in the CAARUD (Centre d’accueil et d’accompagnement à la réduction des risques pour les usagers de drogues). However, special attention is required for those furthest away from the CAARUD, e.g. women, young people, prisoners, migrants, rural residents, slammers, etc. through adapted programs created for these vulnerable groups. Recent data has shown that, on average, a third of PWID visiting CAARUD (75,000 in 2014) in French urban areas had difficulty getting new syringes in the previous 6 months [7]. Nevertheless, the CAARUD still form the backbone of NEP in France with nearly 7 million syringes distributed every year compared with fewer than 5 million in pharmacies [38]. Therefore, it is crucial to continue to develop the CAARUD since they provide access to information, education about safer injection and provide injection, inhalation and snorting equipment.

As the Amsterdam study demonstrated [39], combining OST with integrated harm reduction interventions like NEP and allowing safer injection practices can indeed have a beneficial effect on the reduction of HCV transmission. Moreover, OST contributes indirectly to harm reduction by increasing access to care and encouraging better compliance [40]. It has been shown that OST is associated with a 50% reduction in the risk of new HCV acquisition, and this effect is increased to 74% by the concomitant use of clean drug injecting equipment [31, 41]. The premature discontinuation of HCV treatments is more frequent among “active” PWID who are currently injecting and not enrolled in OST. In 2014, around 180,000 people in France were enrolled in OST, of whom 150,000 were seen in an urban medical practice (105,000 with buprenorphine HD, 45000 with methadone), and 50,000 in a CSAPA (30,000 with methadone and 20,000 with buprenorphine HD), as well as 5000 in prison with some patients receiving dual medical care, which is why the total number is greater than 180,000 (Brisacier, 2015).

A complementary approach is education about the safety of injections. The experimental programs in this area like AERLI (accompagnement et education aux risques liés à l’injection), which is equivalent to ITSESI (individually tailored support and education for safer injection), seem very promising [42, 43]. By observing users’ injecting practices, they can be given face-to-face information on safer ways to inject depending on their personal behavior. Moreover, France has recently decided to experiment drug consumption rooms (DCR). Whether fixed or mobile, DCR can be tested in different sites and with high-risk marginalized populations like “squatters” and recent immigrants, especially those from Eastern Europe. AERLI can thus be given in DCR. The Health Decree of January 27, 2016 authorized the implementation of three experimental DCR in France for the next 6 years. The first two opened in 2016 in Paris and Strasburg, to be followed by Bordeaux in 2019. However, more than three DCR will be needed in the future.

### Testing for HCV infection

Routine HCV testing is essential as it functions on two levels: first, it reduces HCV incidence as knowing one’s status helps to reduce risky behaviors [44, 45]; second, it makes it easier to locate people who could benefit from treatment, which is a another way to reduce HCV prevalence and therefore transmission. To date, many PWID have not been screened including recent injectors, PWID from vulnerable groups, but also occasional users or ex-users who are often socialized, not in touch with a CSAPA or a CAARUD or no longer, and for whom general practitioners end up being the main link to care. Additionally, an investigation carried out in several CAARUD and CSAPA indicated that although more than 9 PWID out of 10 are screened during their lifetime, one third and especially the recent ones are unaware of their HCV positivity [46]. The people and centers who are in contact with PWID, as well as the treatment providers, harm reduction centers, and social workers must therefore do more to increase screening to reach the patients who could be treated with DAA. More outreach with mobile teams is needed wherever users gather, including squats, festivals, etc. Therefore, promoting provider-initiated testing and counselling (PITC) (WHO, 2007) in the prevention or care centers where PWID gather is essential (Morano, 2016).

AERLI has helped to increase uptake of HCV testing [43, 47]. The new rapid testing methods are therefore valuable. In 2014, the French Health Authority (HAS, Haute Autorité pour la Santé) approved the use of a rapid HCV test as part of its HCV screening strategy [48]. To improve screening, it is important to combine counselling strategies with targeted tracking, POCT, outreach and rapid non-invasive tests. They are easy to implement and can reach many PWID [49], in particular the most marginalized. For example, the new molecular testing kits that give a reading of HCV load by a simple hair sample and a drop of blood taken from the finger are very promising [50]. General practitioners also have a crucial role to play when it comes to another “hidden” population, i.e. ex-drug users or “socialized” PWID, who have potentially been contaminated for a long time but have moved away from the drug world (and therefore the specialized centers), who are now socially well-adjusted and are likely to go to a doctor’s office. The general population who may be in touch with “hidden” users should also be made aware of how treatments have changed and of the need for PWID to be treated without stigma. Many ex-PWID, including some who are infected with HCV, challenge and refuse testing and/or treatment as a denial of the past or because they fear former treatments that were not well tolerated and which had little effect on them. This fear is often based on ignorance of current therapeutic progress and the new protocols, so it is important that they should be known about widely.

The data concerning screening frequency is limited, but given the high incidence of HCV infection among PWID in Europe [51] and the great benefit that may be expected, at least one annual screening is recommended for all PWID [52] and a screening every 6 months for active injectors, i.e. at least one injection in the current month. On the other hand, once contamination has occurred, HCV morbidity and mortality can only be reduced by treatment [17].

### Accessing HCV care

Various studies summarized in a recent review [49] demonstrate the benefit of providing DAA treatment at sites used by PWID. People living far away from hospitals should also have access to the new treatments. The teams should adapt their offer to these new patients living furthest away from care settings and provide external help through mobile programs, particularly in rural areas [53] and at GPs’ offices [54].

### Eligibility for care

In the past, HCV treatments were not recommended for active PWID or they were deferred and debated [11]. However, current data show that PWID benefit considerably from DAA because they have a higher risk of HCV transmission and often have a sustained viral response (SVR) when given adapted comprehensive care [49, 55] and are enrolled in an OST program [56]. Therefore, treatment is recommended in France for all infected PWID regardless of their fibrosis level. The goal is to obtain an SVR (undetectable HCV RNA 3 months post-treatment). Studies of classical treatments (pegylated interferon-ribavirin) show equivalent rates of SVR with PWID including active injectors and other patients [57–59]. Treatment by DAA without interferon of a shorter duration is very well tolerated and effective, and major benefit can be expected, in particular in terms of preventing HCV transmission [18, 21, 26, 58]. Therefore, one of the best strategies to reduce HCV transmission among PWID is to treat them. DAA clinical trials classically exclude patients currently injecting, but they sometimes include phase 2 and phase 3 OST patients. In this population, the reinfection rate after viral eradication is lower than that of the incidental rate, and the SVR rates among OST patients appear to be equivalent to those found in other patients [60–62]. No studies to date have reported problematic interactions between DAA and OST and adjustments of OST dosages do not seem necessary [41, 61, 63–66]. Moreover, a recent study found that the longer the duration of OST (methadone), the better the capacity to continue to receive HCV treatment and the better the SVR rates [54]. Lastly, concomitant alcohol consumption does not compromise the effectiveness of the treatment, so it is not contraindicated, even though patients should be encouraged to reduce their consumption.

All these data led the health authorities to radically change their policy, going from injection drug use being a criterion for exclusion [67] to making it a primary indication in terms of public health objectives [25, 52, 60, 68]. Therefore in June 2016, the HAS officially declared that “in an integrative approach to control the HCV epidemic, the patients at highest risk of virus transmission, drug users and others likely to disseminate HCV infection should be able to benefit from these new DAA treatments, regardless of their level of hepatic fibrosis” [69]. This position was endorsed in their recommendations published December 2016 following the expert report that same year and by the new professional recommendations of the French Hepatology Society (AFEF) published in March 2018. These recommendations are supported by the Francophone Infectious Pathology Society (SPILF), which incorporated the simplified therapeutic recommendations made possible by the new pangenotypic treatments (http://www.afef.asso.fr/ckfinder/userfiles/files/recommandations-textes-officiels/recommandations/VF%20INTERACTIF-%20RECO-VHC%20AFEF.pdf) and by the latest European guidelines (EASL 2018, 10.1016/j.jhep.2018.03.026) [41].

### Treatment initiation

Therapeutic pre-evaluation should be multidisciplinary to provide the most comprehensive care including addiction treatment like OST, harm reduction awareness, hepatic care, psychological support and social services. Psychosocial interventions that are designed for patients, their entourage and health professionals can facilitate the initiation of HCV treatment by creating the framework for an integrated approach, particularly if the patient has psychiatric comorbidities [12, 56, 70]. PWID are more likely to adhere to HCV treatment if they are introduced to them by their entourage, most likely social workers or peers, in settings where their drug addiction problems are understood [71, 72]. HCV infection is a chronic liver disease with a risk of cirrhosis. Therefore, patients should be offered non-invasive evaluation of liver fibrosis with blood markers such as the FibroTest or liver stiffness measurement like the FibroScan. In the event of cirrhosis, abdominal ultrasonography is needed every 6 months for hepatocellular carcinoma screening, even after a sustained virologic response is obtained.

### Treatment adherence and compliance

In principle, compliance with DAA should be easier than with the former treatments: better tolerance, fewer pills to take, simpler and less restrictive treatment, shorter duration of treatment. Adherence is much better if the motivation to initiate treatment has been strengthened. Supervised administration of medication can also improve compliance [56], in particular in OST programs [73]. Other studies have shown that compliance is also improved by multidisciplinary approaches [74] involving nurses [75] and psycho-educational techniques [76].

### Integrated care

Experience with AIDS has shown that the quality of addiction treatment and its associated disorders is a key factor in patients’ involvement in their treatment, as it improves compliance at a level similar to that in non-HCV patients [77, 78]. Drug services and hepatic care should be located in the same place. Therefore, the CSAPA and CAARUD provide a full range of care to patients. Although their technical infrastructure may be limited, these frontline facilities contribute to improving screening rates and encouraging patients to attend consultations regularly, as well providing access to hepatic care. Some offer full liver examinations onsite, a factor which contributes to better compliance [79]. They also have a favorable impact on treatment and the prevention of recontamination. They are essential components of the harm reduction arsenal, because their approach is multidisciplinary [80].

The comprehensive “one-stop shop” offered in the CSAPA and CAARUD consists of global HCV testing and treatment, [81], hepatologic consultation, treatment initiation and follow-up. Once a cure is achieved, follow-up is annual. In the event of cirrhosis, it is performed every six months and should include sonographic screening for hepatic carcinoma.

### Prevention of re-infection: harm reduction as a comprehensive intervention [82, 83]

Unlike a relapse, recontamination is a new HCV infection resulting from continued risky injection practices or resumption of drug injection use, despite a cure of the previous infection. Prevention of re-infection is based mainly on the strategy as mentioned above, including the combination of OST and NEP. The risk of re-infection is a major issue as new contamination can limit the preventive effect of treatment. It can also have a negative psychological impact on patients themselves, as some professionals may be reticent to prescribe future access to treatments. However, recent data from seven studies [84] including the Amsterdam cohort study [85] is reassuring, highlighting the relatively low rates of re-infection in treated PWID ranging from 0.8 to 4.7% person-year. These relatively low rates may be due in part to the conditions of the former treatments (pegylated interferon-ribavirin) which required a well-selected compliant population receiving a high-level intervention [85].

The question arises, however, as to the outcomes of people living far from the treatment centers, who do not really request treatment or even have a clinically identified pathology, are potentially more unstable and marginal, and are still injecting. One can assume that they will be at high risk of re-infection, so harm reduction interventions will likely require a specific module for preventing re-infection. As stated by Andrew Ball [82, 86], drug use is associated with multiple and changing health risks and harms that require diverse and complex responses. In an integrated approach, harm reduction comprises a set of interventions that can be adapted to various places and to people with specific needs. A harm reduction package could combine DAA, OST, NEP, ITSESI, fixed and mobile DCRs, psychosocial support and peer interventions as well as an advocacy component for social changes aiming at reducing the stigma surrounding PWID. The impact of HCV treatments is increased when they are combined with prevention, harm reduction interventions and care. Similarly, an increase in the positive effects of HIV and HCV treatments has been observed when they are associated with better access to drug treatment and social services [56, 87, 88]. Peer involvement is also an essential asset, as demonstrated by the web platform “Hepatitis”, managed in France by a PWID support group (Auto-support usagers de drogues, ASUD, http://www.asud.org/hepatite-c/). Support from the Aides Federation has also been crucial to cope with the emerging HCV epidemic in HIV-positive men who have sex with men, where a particularly high level of reinfection seems to exist according to the NEAT data (European AIDS Treatment Network).

Therefore, harm reduction, treatment and prevention are complementary: harm reduction interventions promote access to treatment and healthcare reinforces the effectiveness of harm reduction [89]. The maximum effect is therefore obtained by a combined approach associating DAA and harm reduction (OST and NEP) [21] in a continuum of care and prevention [68].

### Exceptional funding

Universal access to the new HCV treatments is costly. Approximately 750 million € have been invested in HCV each year since 2015, which made it possible to treat nearly 15,000 patients per year. At this pace, the epidemic in France could be eradicated in about ten years. To limit this considerable burden on public finances, the government has set up a unique tax mechanism, named W, which fixes a ceiling on industrial profits. If the expenditure generated by treatments exceeds this budget (a reference amount W), the surplus would be taxed at 100% so no additional public expenditure would be required. In addition, very strict price negotiations have made it possible to obtain less exorbitant drug prices, given the arrival of new molecules on the market which circumvented Gilead’s monopoly with Sovaldi®. In April 2017, new prices were fixed: under 28,700 €, versus 41,000 € previously. A similar agreement has been reached for the pangenotypic medications which were launched in 2017 and 2018 (Epclusa® and Maviret®). Simplified, pangenotypic anti-HCV treatment recommendations are now possible [41]. Furthermore, provisions concerning numbers have been made. If the number of patients being treated were to increase, which is the public health goal, the cost per unit would decrease accordingly. This would allow better control of overall expenditure. The W mechanism should not need to be triggered, nor should prices need to be fixed. However, if necessary, these deterrents would remain available. With this financial framework in place, the strategy proposed in the expert report and the HAS recommendations of December 2016 that followed it, it would be possible to reimburse DAA fully in France. Furthermore, it was decided in March 2018 that the new pangenotypic drugs could henceforth be delivered in pharmacies and no longer only in hospitals.

The next step will be to increase the number of prescribing physicians, which is currently limited to hospital gastroenterologists, hepatologists, and specialists of internal medicine and infectious diseases. To move towards the recommendations of the expert report and to be able to implement the ministry’s decision, the abovementioned treatment strategy should be able to be prescribed not only by these specialists but also by the clinicians working in the CSAPA and CAARUD and by general practitioners who are members of the hepatitis networks. Such a measure is likely to be taken in May 2018.

## Conclusion

Major decreases in prevalent HCV infections occur only when treatment is initiated at the early stages of fibrosis, suggesting that systematic treatment of PWID would be beneficial. However, elimination of HCV within the next 10 years will be difficult to achieve by treatment alone, even with a much-improved offer of care. The new HCV treatments make it possible to treat all patients effectively as well as to envisage the eradication of HCV infection by treating all infected PWID. To achieve elimination of HCV and to avoid HCV reinfection, it is also crucial to strengthen and extend harm reduction policies. These public health objectives require an outreach to people with severe psychosocial vulnerabilities, who often live far from care facilities or harm reduction services. The programs should be adapted to facilitate access to testing and treatment, and to support the monitoring and prevention of reinfection. The present recommendations are based on the international consensus and data showing that treating PWID is easily achievable and effective. This should contribute to making treatment efficient and available throughout France. This should lead to meeting the national objective set by the government of eliminating hepatitis C by 2025 in accordance with the WHO strategy [2, 3].

## Recommendations

1. PWID should be routinely tested for HCV every 12 months and active PWID (at least one injection during the past month) every six months.
2. Given its benefits on the reduction of transmission, treatment is recommended for all PWID with chronic HCV infection.
3. Cessation of injection is not a requirement of HCV treatment.
4. PWID should receive anpre-therapeutic HCV assessment which includes a multidisciplinary evaluation of psychosocial status and drug and alcohol use, allowing for an integrated follow-up.
5. PWID should receive individualized HCV treatment in a clinical setting enabling access to a multidisciplinary team including drug and alcohol services, as well as psychiatric and social services, if possible in a single location.
6. Harm reduction information and counselling should be provided during HCV treatment to prevent HCV re-infection after a successful treatment, especially with PWID who exhibit ongoing risk-behaviors.
7. Following SVR, screening for HCV re-infection in PWID should be performed yearly with HCV RNA.

This research did not receive any special grant from funding agencies in the public, commercial or not-for-profit sectors.

## Footnotes

1 Jean-Michel Delile (coordinator), Xavier Aknine, Antoine Bachelard, Georges Brousse, Michel Doffoel, Stéphanie Dominguez, Juliette Foucher, Marie Jauffret-Roustide, Martine Lacoste, Pascal Mélin, Sébastien Mouveroux, Fabrice Olivet, Brigitte Reiller, Perrine Roux, Gilles Rozsypal,
